# A fresh look at preoperative body washing

**DOI:** 10.1177/1757177411428095

**Published:** 2012-01

**Authors:** Judith Tanner, Dinah Gould, Philip Jenkins, Rachel Hilliam, Neetesh Mistry, Susannah Walsh

**Affiliations:** 1. Faculty of Health and Life Science, De Montfort University, The Gateway, Leicester, LE1 9BH, UK; 2. School of Health Sciences, City University, London, UK; 3. Faculty of Art and Design, De Montfort University, Leicester, UK; 4. Research and Development, Derby Hospitals NHS Foundation Trust, Derby, UK; 5. Faculty of Health and Life Science, De Montfort University, Leicester, UK

**Keywords:** Preoperative body washing, chlorhexidine gluconate, octenidine

## Abstract

National guidelines do not support preoperative body washing to reduce surgical site infections, instead recommending bathing or showering with soap. Yet preoperative body washing continues to be widely used in many hospitals across Europe. This paper suggests that existing trials of preoperative body washing, upon which guidelines are based, are dated and proposes a new investigation of preoperative body washing using modern definitions of surgical site infection with standardised patient follow up, modern surgical techniques and well designed trials. This paper provides a critique of existing guidelines and describes a randomised trial with 60 participants to compare the effect of soap and two antiseptic washing products on colony forming units (CFUs) for up to six hours. Chlorhexidine gluconate and octenidine were significantly more effective than soap in reducing CFUs in the underarm, and chlorhexidine was significantly more effective than soap in reducing CFUs in the groin.

## Introduction

The concept of preoperative body washing; washing with an antiseptic solution before surgery, was introduced over 30 years ago with the aim of reducing skin bacterial load and ultimately reducing endogenous surgical site infections (SSIs) (Cruse and Foord, 1980). Despite guidelines (NICE, 2008) and systematic reviews (Webster and Osborne, 2011) that do not support this practice, preoperative body washing is routine in some hospitals in Europe, especially in the UK, Sweden and the Netherlands. It is unusual for a clinical intervention with an associated cost to continue to be widely used in contradiction to national guidelines. This situation calls for a reappraisal of the effectiveness of preoperative body washing.

## Literature review

Probably the two most influential documents in the UK which discuss preoperative body washing are the SSI guidelines published by the National Institute for Health and Clinical Excellence (NICE, 2008) and the Cochrane systematic review of body washing (Webster and Osborne, 2011). Based upon a review of the evidence neither of these documents recommends preoperative body washing. The NICE guidelines recommend that patients shower or bathe using soap either the day before or the day of surgery and the Cochrane review found no clear evidence to support or reject preoperative body washing. However, examination of the evidence included in these documents identifies some potential concerns. For example, the NICE recommendation is based on a meta analysis of five trials published between 20 and 29 years ago (Byrne et al, 1992; Earnshaw et al, 1989; Hayek et al, 1987; Randall et al, 1983; Rotter et al, 1988). The age of the trials is important as they pre-date standardised definitions of SSIs and surveillance protocols and developments within clinical practice, such as the introduction of routine antibiotic prophylaxis (Horan et al, 1992). It is interesting to note the comparatively high SSI rates in the included trials, ranging from 10%–50%.

Of further interest is that while this meta-analysis led NICE to recommend the use of soap, only three (Earnshaw et al, 1989; Hayek et al, 1987; Randall et al, 1983) of the five studies included in the meta-analysis used soap as a comparison group, with the remaining studies comparing an antiseptic agent against a detergent. Additionally, one study (Hayek et al, 1987), which compared soap, detergent and an antiseptic was flawed. In the flawed study it was subsequently discovered that the ‘detergent’ had antimicrobial properties, yet the contaminated data from patients receiving the ‘detergent’ is still included in the meta-analysis.

The same five trials are included in the Cochrane systematic review of preoperative bathing or showering, although they are separated into trials comparing antiseptic solutions against soap and trials comparing antiseptic solutions against detergent. Three trials (Earnshaw et al, 1989; Hayek et al, 1987; Randall et al, 1983) are included in the meta-analysis comparing antiseptic solutions (chlorhexidine gluconate) against soap. One trial from 1983 with 62 participants and a patient follow up of seven days found no difference in SSI rates (Randall et al, 1983). A second trial from 1989 with 66 participants and a patient follow up of around five days found in favour of soap (Earnshaw et al, 1989). The third trial with 1315 patients followed up for six weeks (Hayek et al, 1987). This large trial established superior performance for chlorhexidine with an infection rate in the chlorhexidine group of 9% compared to 13% for the soap group. However, this statistical significance was lost when the trial was combined in a meta-analysis with the two small studies using a random effects model (*p* value 0.94), notably heterogeneity remained high (I^2^ =60%).

The limitations of these trials suggests that a new study using modern definitions, modern clinical practice and high quality design is required.

We therefore proposed a two stage study which would address the deficits of the existing studies and allow a comparison of soap against antiseptic agents. The first stage was to identify the effect of preoperative body washing on skin bacterial counts and the second stage was to identify the effect of preoperative body washing on surgical site infections. Splitting the study into two stages was a necessary step for ethical reasons. If a randomised trial of body washing showed no difference in skin bacterial counts, then it would be pointless to conduct a large expensive trial involving patients to measure SSI rates. Conversely, if a trial of body washing showed a difference in skin bacterial counts in favour of antiseptics then a trial measuring SSIs would be justified. This paper describes the first stage of the study.

## Aim of the study

The aim of the first stage of the study reported in this paper was to measure the immediate and residual effect of two body washing antiseptic products against soap with colony forming units as the outcome measure.

## Materials and methods

We undertook a randomised controlled trial with plain soap, chlorhexidine gluconate and octenidine comparing the reduction in colony forming units in 60 healthy volunteers.

Following ethical approval from the University Research Ethics Committee and with consent, 60 participants were randomised to shower with one of the following products;

- Plain liquid soap (Morrisons)
- HiBi Scrub Plus (4% w/v chlorhexidine gluconate, Molnlycke Health Care)
- Octenisan (1% octenidine dihydrochloride, Schulke)

In accordance with manufacturers’ instructions, chlorhexidine gluconate was used once a day for two consecutive days and octenidine was used once a day for five consecutive days. Body contact time for chlorhexidine gluconate was one minute compared to three minutes for octenidine. As there were no manufacturers’ instructions for plain soap we matched the soap application method with the chlorhexidine method. The method of application was also taken from the manufacturers’ instructions.

### Sample size

A pragmatic decision was taken to recruit 60 participants. Volunteers were recruited until 60 participants completed the study. Drop out rates are reported and missing data is accounted for.

### Recruitment method and exclusion criteria

University students and staff volunteers were recruited through flyers. Volunteers who responded to recruitment and met the inclusion and exclusion criteria were accepted onto the study. Inclusion criterion was access to a shower. Exclusion criteria were as follows:

- Allergies to ingredients in the soap, HiBi Scrub Plus or Octenisan products
- Open wounds
- Antibiotics currently or in previous week
- Respiratory infection
- Skin infection
- Jewellery in nose

### Risk of bias

Randomisation sequence was generated using block randomisation with multiples of three. Group allocation details were placed inside sequentially numbered sealed envelopes. The researchers overseeing baseline data collection were aware of each participant’s group allocation status, but laboratory staff were blinded to this.

### Outcome measures

Anonymous demographic data were collected to validate the randomisation. Baseline swabs were taken before body washing commenced, immediately after the final shower and then again at four hours and six hours. Swab samples were taken from the nostrils, underarms and groin. Rayon tipped swabs were pressed firmly against the skin and rotated. At the end of the study, participants were also asked if the product had caused any skin conditions, such as redness, itchiness or dryness.

## Laboratory methods

Before the study commenced, each stage of bacterial sampling method was tested in the laboratory. This included bacterial controls, swab selection, neutraliser tests and the study sampling protocol.

### Controls

Control experiments using a mixed culture of *Staphylococcus aureus* ATCC 6538 (*S. aureus*), *Staphylococcus warneri* NCTC 7291 (*S. warneri*) and *Escherichia coli* ATCC 10536 (*E. coli*) were conducted to validate the test methods. Experiments were performed independently on at least three occasions.

### Swab selection

Recovery of micro-organisms from Amies charcoal transport swabs (Technical Services Consultants) was compared to use of Sterilin rayon tipped plain sterile swabs (Fisher Scientific) and neutraliser. Recovery of micro-organisms using the rayon tipped swab and neutraliser method was equivalent to that achieved with charcoal media, so this method was adopted for the study as it was more cost effective.

### Validation of non-toxicity of neutraliser

The purpose of this test was to show that the solution in which swabs were transported did not kill any bacteria present on the swabs. Monocultures of *S. aureus, S. warneri* and *E. coli* were grown overnight (18 hours) at 37ºC in 10 mL of casein soya bean digest broth (Oxoid) in an incubator shaking at 100 rpm. The overnight cultures were centrifuged at 4000 rpm for 10 minutes and resuspended in 10 mL of sterile phosphate buffered saline (PBS) to remove the culture media. 5 mL of each organism were pooled together to make 15 mL of mixed culture test suspension.

Sterilin rayon tipped swabs were dipped into the mixed culture test suspension and pressed gently against the side to the container to remove excess. The tip of each swab was cut off using scissors into a universal containing 10 mL of either sterile neutraliser or PBS. This inoculated neutraliser and PBS was stored at room temperature for one hour (to simulate the maximum transportation time to the laboratory) and then at 4ºC for a further two hours (three hours storage in total). Universals were vortexed briefly, then aliquots were taken at zero, one, two and three hours and serially diluted in PBS before enumeration using the spread plate technique on casein soya bean digest agar (Oxoid). The neutraliser was not toxic to the control micro-organisms over a three hour period (one hour at room temperature and two hours at 4ºC), giving similar survival to organisms in phosphate buffered saline under the same conditions.

### Validation of neutraliser efficacy

The purpose of this test was to show that the solution in which swabs were transported was effective in preventing any product (soap, HiBi Scrub Plus or Octenisan) from continuing to act on bacteria. 0.1 mL of each test product was added to 9 mL of sterile neutraliser and 0.9 mL of sterile distilled water to give a final concentration of 30g/L tween 80 and 3g/L soya bean lecithin. The solution was vortexed to mix thoroughly and then immediately inoculated with a swab that had been dipped in a mixed culture test suspension (as detailed previously). The inoculated universals containing neutraliser, product mixture and bacteria were stored at room temperature for one hour (to simulate the maximum transportation time to the laboratory) and then at 4ºC for a further two hours (three hours storage in total). Universals were vortexed briefly, then aliquots were taken at zero, one, two and three hours and serially diluted in PBS before enumeration using the spread plate technique on casein soya bean digest agar. An aqueous solution of 30 g/L tween 80 (Fisher Scientific) and 3 g/L soya bean lecithin (BDH) in PBS dubecco A pH 7.3 (Oxoid), was effective in neutralising the activity of 1 in 100 dilutions of all test products. This dilution factor is far less than the predicted in-use dilution, assuming that volunteers use 30 mL of product and shower for five minutes using 17.5 L of water (data obtained from the Environment Agency for a low performance 7.2 kW electric shower).

### Laboratory test protocol

Neutraliser solutions containing the rayon tips of volunteer swabs were transported to the microbiology laboratory and refrigerated to 4ºC within one hour of sample collection. Aliquots were taken within the validated three hour time limit and serially diluted in PBS before enumeration using the spread plate technique on casein soya bean digest agar. All Petri-dishes were incubated for 72 hours at 37ºC, as preliminary testing showed that the number of colonies present on the agar reached a maximum at this time point. The number of colony forming units originally present on the volunteers’ swabs was then calculated. Because of the limit of sensitivity of the spread plate method, the minimum number of colony forming units that could be detected was 50 per swab.

### Statistical analysis

A repeated measures ANOVA was used to compare the difference in average log reduction of colony forming units per swab between the three groups from pre-intervention to the three post-intervention time points. These tests were carried out separately for the three swab sites.

## Results

Sixty participants completed the study over a period of two months. One participant failed to complete the final swab sample and was replaced. The laboratory results for two participants were contaminated and these participants were also replaced. None of the participants reported any skin irritations.

### Participant demographics

The *p* value for the chi-squared statistic showed a similar distribution by gender within each group (0.928) and there was no evidence of a difference in age distribution (*p* 0.564).

### Analysis for underarm

**Figure 1.**
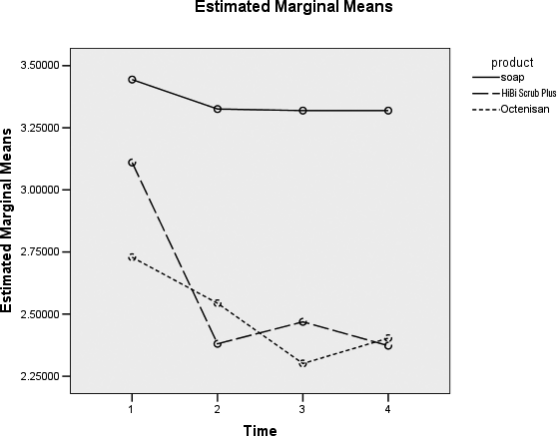
Difference between product type over time for underarm.1 = Baseline, 2 = 0 hours post-intervention, 3 = 4 hours post-intervention and 4 = 6 hours post-intervention

Analysis of Figure 1 shows there is a significant difference between the products (*p*=0.007) (not shown). These differences are evident in Table 1 with a significant difference between soap and HiBi Scrub Plus (*p*=0.011) and also between soap and Octenisan (*p*=0.004). In summary for the underarm, there is significant evidence of a difference in log counts between soap and the two antiseptic products, and the drop in counts is significant at hours four and six (*p*=0.024) (not shown).

**Table 1.** Differences between products for underam

Contrast results (K Matrix)			
Product Simple Contrast ^a^			Averaged Variable
			MEASURE_1

HiBi Scrub Plus vs. soap	Contrast estimate		−0.769
	Hypothesized value		0
	Difference (estimate - hypothesized)		−0.769
	Std. Error		0.291
	Sig.		0.011
	95% confidence interval	Lower Bound	−1.351
	for difference	Upper Bound	−0.186
Octenisan vs. soap	Contrast estimate		−0.858
	Hypothesized value		0
	Difference (estimate - hypothesized)		−0.858
	Std. Error		0.284
	Sig.		0.004
	95% confidence interval	Lower Bound	−1.426
	for difference	Upper Bound	−0.290

a. Reference category = soap

### Analysis for groin

**Figure 2.**
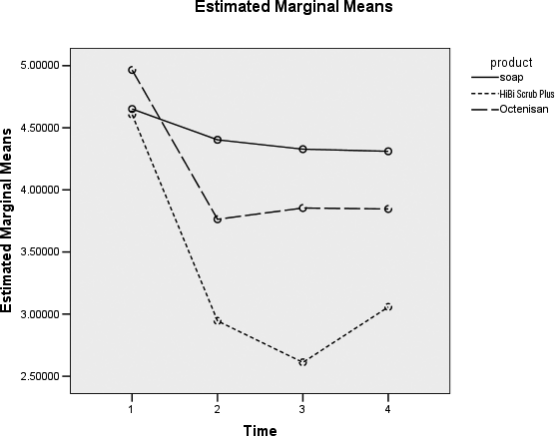
Difference between product type over time for groin. 1 = Baseline, 2 = 0 hours post-intervention, 3 = 4 hours post-intervention and 4 = 6 hours post-intervention

Figure 2 shows the differences between soap, HiBi Scrub Plus and Octenisan. These differences are shown in Table 2 with a significant effect between soap and HiBi Scrub Plus (*p*=0.000) but not between soap and Octenisan (*p*=0.212). In summary for the groin, there is significant evidence of a difference in log counts between HiBi Scrub Plus and the other two products, and the drop in counts is significant at hours four and six (*p*=0.000) (not shown).

**Table 2.** Differences between products for groin

Contrast results (K Matrix)			
Product Simple Contrast ^a^			Averaged Variable
			MEASURE_1

HiBi Scrub Plus vs. soap	Contrast estimate		−1.118
	Hypothesized value		0
	Difference (estimate - hypothesized)		−1.118
	Std. Error		0.256
	Sig.		0.000
	95% confidence interval	Lower Bound	−1.632
	for difference	Upper Bound	−0.604
Octenisan vs. soap	Contrast estimate		−0.316
	Hypothesized value		0
	Difference (estimate - hypothesized)		−0.316
	Std. Error		0.250
	Sig.		0.212
	95% confidence interval	Lower Bound	−0.817
	for difference	Upper Bound	0.185

a. Reference category = soap

### Analysis for nose

**Figure 3.**
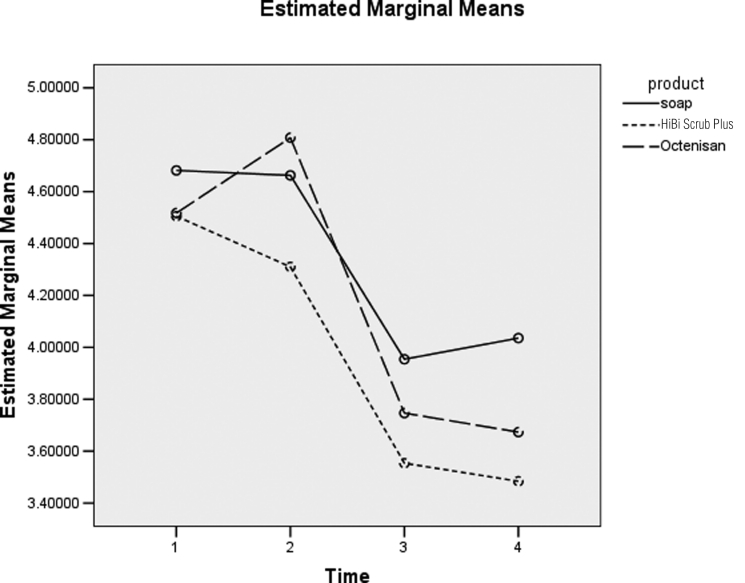
Difference between product type over time for nose. 1 = Baseline, 2 = 0 hours post-intervention, 3 = 4 hours post-intervention and 4 = 6 hours post-intervention

Figure 3 does not show enough evidence for a difference in product types (*p*=0.458) (not shown). There is not enough evidence to suggest a difference in log counts between the three products.

## Limitation

A limitation of this study was that participants took their own swab samples although this was conducted after a training session from the researcher and it was not possible to check participants’ showering technique.

## Discussion

New studies using rigorous methods of SSI surveillance for 30 days are identifying infection rates which are considerably higher than existing data (Tanner et al, 2009). This, in conjunction with the high cost of SSIs, has led to a renewed focus on interventions to reduce surgical site infections (House of Commons Public Accounts Committee, 2009). Preoperative body washing is a relatively inexpensive intervention and therefore should be assessed properly before it is dismissed as having no additional benefit over soap.

This small well conducted trial shows that preoperative body washing products, especially chlorhexidine, are more effective than soap in reducing colony forming units on the skin of healthy volunteers. This effect is seen immediately after showering and also six hours after showering. Chlorhexidine was found to be more effective than octendine, however this was just in the groin. Though the recommended five day application for Octenisan compared with just two days for HiBi Scrub Plus may make this product less appealing to patients.

We are not aware of any studies which demonstrate the relationship between colony forming units and surgical site infections. However, the effect of body washing on colony forming units observed in this study means the possibility exists for body washing to have an effect on surgical site infections. The second stage of this investigation is to conduct a randomised controlled trial among surgical patients comparing soap against a preoperative body wash with antiseptic and measuring SSI as the outcome.

To provide definitive level 1 evidence which can be incorporated into preoperative body washing guidelines a meta-analysis of several clinical trials is needed. To be eligible to be included in a meta-analysis, clinical trials should meet certain key requirements. These are listed below.

- Use showering rather than bathing (as per manufacturers’ instructions)
- Use defined washing protocols – application method, number of applications
- Use standard definitions of surgical site infections
- Be conducted among a single surgical group of patients
- Follow the CONSORT statement when disseminating results to ensure high quality trial reporting (Moher et al, 2001)
